# Socio-demographic and behavioural determinants of weight gain in the Swiss population

**DOI:** 10.1186/s12889-015-1451-9

**Published:** 2015-01-31

**Authors:** Filipa Guerra, Silvia Stringhini, Peter Vollenweider, Gérard Waeber, Pedro Marques-Vidal

**Affiliations:** Faculty of Medicine, University of Lisbon, Lisbon, Portugal; Institute of Social and Preventive Medicine, Lausanne University Hospital, Lausanne, Switzerland; Department of Internal Medicine, Lausanne University Hospital (CHUV), Rue du Bugnon 46, Lausanne, 1011 Switzerland

**Keywords:** Weight gain, Socio-demographic, Prospective study, Switzerland, Population-based

## Abstract

**Background:**

In Switzerland, socio-demographic and behavioural factors are associated with obesity, but no study ever assessed their impact on weight gain using prospective data.

**Methods:**

Data from 4,469 participants (53.0% women), aged 35 to 75 years at baseline and followed for 5.5 years. Weight gain was considered as a rate (kg/year) or as gaining ≥5 kg during the study period.

**Results:**

Rate of weight gain was lower among participants who were older (mean ± standard deviation: 0.46 ± 0.92, 0.33 ± 0.88, 0.21 ± 0.86 and 0.06 ± 0.74 kg/year in participants aged [35-45], [45-55], [55–65] and [65+] years, respectively, P<0.001); physically active (0.27 ± 0.82 vs. 0.35 ± 0.95 kg/year for sedentary, P < 0.005) or living in couple (0.29 ± 0.84 vs. 0.35 ± 0.96 kg/year for living single, P < 0.05), and higher among current smokers (0.41 ± 0.97, 0.26 ± 0.84 and 0.29±0.85 kg/year for current, former and never smokers, respectively, p<0.001). These findings were further confirmed by multivariable analysis. Multivariable logistic regression showed that receiving social help, being a current smoker or obese increased the likelihood of gaining ≥5 Kg: Odds ratio (OR) and 95% confidence interval (CI) 1.43 (1.16-1.77); 1.63 (1.35-1.95) and 1.95 (1.57-2.43), respectively, while living in couple or being physically active decreased the risk: 0.73 (0.62-0.86) and 0.72 (0.62-0.83), respectively. No association was found between weight gain and gender, being born in Switzerland or education.

**Conclusions:**

In Switzerland, financial difficulties (indicated by receiving social help) and current smoking were associated with increases in body weight over a 5 years follow-up. Living in couple, being older or physically active were protective against weight gain.

**Electronic supplementary material:**

The online version of this article (doi:10.1186/s12889-015-1451-9) contains supplementary material, which is available to authorized users.

## Background

Worldwide prevalence of obesity almost doubled between 1980 and 2008 [1] and a similar trend has been observed in Switzerland [2]. Several socio-demographic and behavioural factors have been shown to influence weight gain. A consistent positive association between marital status [3], occupational position [4], low educational level, economic difficulties [5] and weight gain has been reported. Still, the impact of SES on weight gain might differ according to gender [6] or to the country’s level of socioeconomic development – while in high income countries a high socioeconomic status (SES) is generally related to a lower prevalence of obesity, the opposite association is found in low income countries [7].

In Switzerland, several cross-sectional studies have shown an inverse association between obesity and socio-demographic and behavioural factors [8,9], but whether socio-demographic and behavioural factors have an impact on weight gain has never been investigated prospectively. Indeed, one of the main objectives of the Swiss national programme on healthy eating and physical activity (PNAAP) is achieving a health weight [10] and such data are important for adequately designing health promotion policies and to evaluate their impact in the target population.

Thus, we aimed to assess the socio-demographic and behavioural determinants of weight gain, using prospective data from the Swiss population-based CoLaus study.

## Methods

### The Cohorte Lausannoise (CoLaus) study

The CoLaus study is a population-based study assessing the clinical, biological and genetic determinants of cardiovascular disease in the city of Lausanne, Switzerland [11]. The initial recruitment took place between June 2003 and May 2006 and enrolled 6,733 participants (3,544 women) aged 35–75 years; participation rate was 41%.

Follow-up was conducted between April 2009 and September 2012 and included all participants of the baseline study willing to participate. At follow-up, participants attended a single visit which included, as in the baseline assessment, an interview, a physical exam, and blood and urine collections in the fasting state. Average follow-up time was 5.5 years.

### Socio-economic data

Educational level was categorized as primary, secondary school, apprenticeship and university. Nationality was defined by the country of birth and categorized into Swiss and the most frequent nationalities in the canton (French, Italian, Portuguese and Spanish) and other. Marital status was categorized into living in couple (married and other relationship) or living alone (single, divorced or widowed).

Receiving social help was assessed with the question: “Do you receive social help?”. Because all individuals residing in Switzerland receive financial compensation when they retire, the response to this variable is not informative beyond the retirement age. Therefore, men older than 65 years and women older than 63 or 64 years were not considered as receiving social help (N = 638). In Switzerland, social help is provided as financial support to people with disabilities or whose income is insufficient to support themselves or their family, and can thus be considered as an indicator of financial adversity.

### Clinical and anthropometric data

Smoking status was defined as never, former and current. Physical activity was self-reported and participants were considered as physically active if they reported practicing leisure time physical activity at least twice per week. Body weight, height and waist circumference (WC) were measured using standard procedures [11]. The same procedure was used in the baseline and follow-up examinations. BMI was defined as weight(kg)/height(m)^2^. Normal BMI was defined as BMI < 25 kg/m^2^, overweight as 25≤BMI<30 kg/m^2^ and obesity as BMI≥30 kg/m^2^. Abdominal obesity was defined as WC≥102 cm for men and WC ≥ 88 cm for women.

Weight gain was considered in two ways. First, as a continuous variable representing the rate of weight gain, computed as the difference between the follow-up and baseline weight divided by the number of years of the follow-up (Kg/year). Second, as having or not having gained 5 Kg or more during the follow-up; the 5 Kg cut-off was chosen according to the recommendations of the World Health Organization that weight gain in adulthood should not exceed 5 Kg over the entire adult life [12].

### Ethics

The study was approved by the Institutional Ethics Committee of the University of Lausanne and all participants provided written informed consent.

### Statistical analysis

Analyses were conducted after excluding participants without BMI at baseline or follow-up, without SES data at baseline. As illness could lead to involuntary weight loss, participants who reported involuntary weight loss during the last 12 months at follow-up were also excluded.

Statistical analyses were performed using Stata version 12.0 for Windows (Stata Corp, College Station, Texas, USA). Descriptive results were expressed as number of participants (percentage) or as mean ± standard deviation. Bivariate analyses were performed using chi-square or Fisher's exact test for qualitative variables and one-way analysis of variance (ANOVA) for quantitative variables. Multivariable analyses were performed using ANOVA or logistic regression and results were expressed as multivariable adjusted mean ± standard error for ANOVA or as Odds ratio (OR) and 95% confidence interval (CI) for logistic regression. In multivariable analyses, several models were considered – adjusted only for age and gender and adjusted for all the variables significantly associated with weight gain. As BMI and waist were correlated, two separate multivariable models were tested (one with BMI and another with waist). Statistical significance was considered for p < 0.05.

## Results

### Characteristics of the participants

Of the initial 5,064 participants with follow-up data, 67 (1.3%) were excluded because of missing values on BMI at baseline or follow-up and 528 (10.4%) because of involuntary weight loss during the last 12 months. The socio-demographic and anthropometric characteristics of the remaining 4,469 participants are summarized in Table 1.

**Table 1:** **Sample characteristics (N = 4,469) at baseline and factors associated with weight gain, excluding participants reporting involuntary weight loss**

	**All participants**	**Weight gain (kg/year)**	**Gain≥5 kg (%)**
**N**	**4,469**		**868**
Age group	N (%)	Mean ± SD	N (%)
[35-45]	1 390 (31.1)	0.46 ± 0.92	334 (24.0)
[45-55]	1 344 (30.1)	0.33 ± 0.88	272 (20.2)
[55-65]	1 178 (26.4)	0.21 ± 0.86	202 (17.2)
[65–75]	557 (12.5)	0.06 ± 0.74	60 (10.8)
p-value comparing groups		<0.001	<0.001
Gender			
Women	2 369 (53.0)	0.31 ± 0.92	461 (19.5)
Men	2 100 (47.0)	0.31 ± 0.84	407 (19.4)
p-value comparing groups		0.90	0.95
Born in Switzerland			
No	1 660 (37.1)	0.34 ± 0.85	340 (20.5)
Yes	2 809 (62.9)	0.29 ± 0.90	528 (18.8)
p-value comparing groups		<0.05	0.17
Nationality			
Swiss	2 809 (62.9)	0.29 ± 0.90	528 (18.8)
French	285 (6.4)	0.35 ± 0.77	54 (19.0)
Italian	238 (5.3)	0.41 ± 0.87	56 (23.5)
Portuguese	226 (5.1)	0.24 ± 0.91	36 (15.9)
Spanish	157 (3.5)	0.36 ± 0.80	34 (21.7)
Other	754 (16.9)	0.35 ± 0.86	160 (21.2)
p-value comparing groups		0.12	0.21
Receiving social help			
No	3 930 (87.9)	0.30 ± 0.86	728 (18.5)
Yes	539 (12.1)	0.37 ± 1.00	140 (26.0)
p-value comparing groups		0.06	<0.001
Marital status			
Living alone	1 414 (31.7)	0.35 ± 0.96	316 (22.4)
Living in couple	3 052 (68.3)	0.29 ± 0.84	552 (18.1)
p-value comparing groups		<0.05	<0.001
Educational level			
Primary	755 (16.9)	0.30 ± 0.98	150 (19.9)
Apprenticeship	1 579 (35.3)	0.29 ± 0.92	324 (20.5)
Secondary school	1 158 (25.9)	0.31 ± 0.86	217 (18.7)
University	977 (21.9)	0.32 ± 0.76	177 (18.1)
p-value comparing groups		0.84	0.44
Smoking status			
Never	1 848 (41.4)	0.29 ± 0.85	312 (16.9)
Former	1 513 (33.9)	0.26 ± 0.84	274 (18.1)
Current	1 108 (24.8)	0.41 ± 0.97	282 (25.5)
p-value comparing groups		<0.001	<0.001
Physical activity			
No	2 018 (45.2)	0.35 ± 0.95	457 (22.7)
Yes	2 451 (54.8)	0.27 ± 0.82	411 (16.8)
p-value comparing groups		0.005	<0.001
Body mass index categories			
Normal	2 192 (49.1)	0.35 ± 0.70	366 (16.7)
Overweight	1 651 (36.9)	0.29 ± 0.85	343 (20.8)
Obese	626 (14.0)	0.22 ± 1.37	159 (25.4)
p-value comparing groups		0.002	<0.001
Abdominal obesity			
No	3 220 (72.1)	0.34 ± 0.74	583 (18.1)
Yes	1 249 (28.0)	0.22 ± 1.16	285 (22.8)
p-value comparing groups		<0.001	<0.001

Results are expressed as average ± standard deviation or as number of participants (percentage). Statistical analysis by chi-square or analysis of variance.

### Determinants associated with weight gain

Bivariate analysis of the baseline factors associated with rate of weight gain or weight gain ≥5 Kg are summarized in Table 1. Rate of weight gain decreased with age, and was lower among participants born in Switzerland, living in couple or physically active. Obese (BMI or abdominal) participants had a lower rate of weight gain, while current smokers had a higher rate. Receiving social help tended to be associated with weight gain (p = 0.06). The factors associated with a weight gain ≥5 Kg were similar, although the association with being born in Switzerland was no longer significant, while receiving social help became significant.

Results of multivariable analyses adjusting for age and gender (model 1) and for the main confounders (model 2) are summarized in Table 2. Weight gain rate was negatively associated with age, living in couple and being physically active, and positively associated with current smoking. Weight gain rate was also associated with nationality (other than Portuguese), while no significant relationship was found with being Swiss born or BMI status. Multivariable logistic regression on gaining ≥5 Kg provided similar results, i.e. that older vs. younger participants and those living in couple vs. alone had, respectively, a 62% (95% CI: 0.28-0.51) and a 27% (95% CI: 0.62-0.86) decreased risk of weight gain, while current vs. never smokers had a 63% increased risk (95% CI: 1.35-1.95). Participants receiving social help had a 43% higher likelihood (95% CI: 1.16-1.77) of gaining ≥5 kg.

**Table 2:** **Multivariable analysis of the factors associated with weight gain (N = 4,469), excluding participants reporting involuntary weight loss at follow-up**

	**Weight gain (kg/year)**			**Gain ≥5 kg**		
	**Model 1**	**Model 2**	**Model 2**	**Model 1**	**Model 2**	**Model 2**
Age group						
[35-45]	0.46 ± 0.02	0.45 ± 0.02	0.47 ± 0.02	1 (ref.)	1 (ref.)	1 (ref.)
[45-55]	0.33 ± 0.02	0.33 ± 0.02	0.33 ± 0.02	*0.80 (0.67 - 0.96)*	*0.76 (0.63 - 0.92)*	*0.74 (0.62 - 0.90)*
[55-65]	0.21 ± 0.03	0.22 ± 0.03	0.21 ± 0.03	*0.65 (0.54 - 0.79)*	*0.60 (0.49 - 0.73)*	*0.56 (0.46 - 0.69)*
[65–75]	0.06 ± 0.04	0.08 ± 0.04	0.08 ± 0.04	*0.38 (0.28 - 0.51)*	*0.37 (0.27 - 0.50)*	*0.35 (0.25 - 0.47)*
p-value between groups	<0.001^a^	<0.001	<0.001	<0.001^a,d^	<0.001^d^	<0.001^d^
Gender						
Women	0.31 ± 0.02	0.31 ± 0.02	0.31 ± 0.02	1 (ref.)	1 (ref.)	1 (ref.)
Men	0.30 ± 0.02	0.31 ± 0.02	0.30 ± 0.02	0.97 (0.84 - 1.13)	0.93 (0.79 - 1.08)	1.02 (0.87 - 1.19)
p-value between groups	0.55^b^	0.88	0.73	0.72^b^	0.34	0.80
Born in Switzerland						
No	0.32 ± 0.02	0.32 ± 0.02	-	1 (ref.)	1 (ref.)	-
Yes	0.30 ± 0.02	0.30 ± 0.02	-	0.97 (0.83 - 1.13)	1.04 (0.89 - 1.22)	-
p-value between groups	0.47	0.52	-	0.66	0.61	
Nationality						
Swiss	0.30 ± 0.02	-	0.30 ± 0.02	1 (ref.)	1 (ref.)	1 (ref.)
French	0.34 ± 0.05	-	0.33 ± 0.05	0.96 (0.70 - 1.32)	-	0.98 (0.71 - 1.34)
Italian	0.45 ± 0.06	-	0.45 ± 0.06	*1.41 (1.02 - 1.93)*	-	1.36 (0.98 - 1.88)
Portuguese	0.13 ± 0.06	-	0.12 ± 0.06	*0.65 (0.45 - 0.95)*	-	*0.60 (0.41 - 0.88)*
Spanish	0.35 ± 0.07	-	0.35 ± 0.07	1.12 (0.75 - 1.66)	-	1.10 (0.74 - 1.65)
Other	0.32 ± 0.03	-	0.31 ± 0.03	1.07 (0.88 - 1.31)	-	1.01 (0.82 - 1.25)
p-value between groups	0.006	-	0.005	-	-	-
Receiving social help						
No	0.30 ± 0.01	0.30 ± 0.01	0.30 ± 0.01	1 (ref.)	1 (ref.)	1 (ref.)
Yes	0.34 ± 0.04	0.33 ± 0.04	0.34 ± 0.04	*1.43 (1.16 - 1.77)*	*1.28 (1.03 - 1.58)*	*1.32 (1.07 - 1.64)*
p-value between groups	0.32	0.49	0.40	0.001	0.03	0.02
Marital status						
Living alone	0.36 ± 0.02	0.36 ± 0.02	0.35 ± 0.02	1 (ref.)	1 (ref.)	1 (ref.)
Living in couple	0.28 ± 0.02	0.29 ± 0.02	0.29 ± 0.02	*0.73 (0.62 - 0.86)*	*0.76 (0.65 - 0.90)*	*0.77 (0.66 - 0.91)*
p-value between groups	0.007	<0.05	<0.05	<0.001	0.001	0.002
Educational level						
Primary	0.32 ± 0.03			1 (ref.)	1 (ref.)	1 (ref.)
Apprenticeship	0.31 ± 0.02	-	-	1.05 (0.84 - 1.30)	-	-
Secondary school	0.30 ± 0.03	-	-	0.88 (0.70 - 1.11)	-	-
University	0.29 ± 0.03	-	-	0.81 (0.63 - 1.03)	-	-
p-value between groups	0.95	-	-	0.02^d^		
Smoking status						
Never	0.29 ± 0.02	0.29 ± 0.02	0.29 ± 0.02	1 (ref.)	1 (ref.)	1 (ref.)
Former	0.28 ± 0.02	0.28 ± 0.02	0.28 ± 0.02	1.14 (0.95 - 1.36)	1.14 (0.95 - 1.36)	1.13 (0.94 - 1.36)
Current	0.39 ± 0.03	0.37 ± 0.03	0.37 ± 0.03	*1.63 (1.35 - 1.95)*	*1.59 (1.32 - 1.91)*	*1.56 (1.29 - 1.88)*
p-value between groups	0.003	<0.05	<0.05	<0.001^d^	<0.001^d^	<0.001^d^
Physical activity						
No	0.34 ± 0.02	0.34 ± 0.02	0.34 ± 0.02	1 (ref.)	1 (ref.)	1 (ref.)
Yes	0.28 ± 0.02	0.28 ± 0.02	0.28 ± 0.02	*0.72 (0.62 - 0.83)*	*0.79 (0.68 - 0.92)*	*0.77 (0.66 - 0.90)*
p-value between groups	<0.05	<0.05	<0.05	<0.001	0.003	0.001
Body mass index categories			-			
Normal	0.33 ± 0.02	0.33 ± 0.02	-	1 (ref.)	1 (ref.)	-
Overweight	0.30 ± 0.02	0.30 ± 0.02	-	*1.46 (1.23 - 1.73)*	*1.43 (1.21 - 1.70)*	-
Obese	0.25 ± 0.03	0.24 ± 0.04	-	*1.95 (1.57 - 2.43)*	*1.89 (1.51 - 2.37)*	-
p-value between groups	0.13	0.08	-	<0.001^d^	<0.001^d^	
Abdominal obesity						
No	0.32 ± 0.02	-	0.32 ± 0.02	1 (ref.)	-	1 (ref.)
Yes	0.27 ± 0.03	-	0.26 ± 0.03	*1.58 (1.34 - 1.87)*	-	*1.52 (1.28 - 1.80)*
p-value between groups	0.08	-	<0.05	<0.001		<0.001

OR: Odds Ratio; BMI: Body Mass Index. Results are expressed as multivariable adjusted mean ± standard error of the mean (sem) or as odds-ratio (OR) and (95% CI). Statistical analysis by analysis of variance or logistic regression. Model 1, adjusting for age and gender, except ^a^adjusted for gender only; ^b^adjusted for age only; Model 2, adjusted for all the variables in the model (indicated in the column); ^d^p-value of the test for trend; −, not included in the model. Statistically significant (p < 0.05) ORs are indicated in italic.

Repeating the same analyses on the whole sample (i.e. including participants who were initially excluded from the analysis) led to similar findings, except that the difference in rate of weight gain between physically active and non-active participants was no longer significant, and that the increased likelihood of gaining ≥5 kg was borderline significant among participants receiving social help (Additional file 1: Table S1).

## Discussion

To our knowledge, this is the first study that assessed the socio-demographic determinants of weight gain in the general Swiss adult population. Our results suggest that weight gain is negatively associated with age, Portuguese nationality, living in couple and physical activity, and positively associated with current smoking, receiving social help and obesity. Conversely, no association between weight gain and educational level was found.

**Figure 1:**
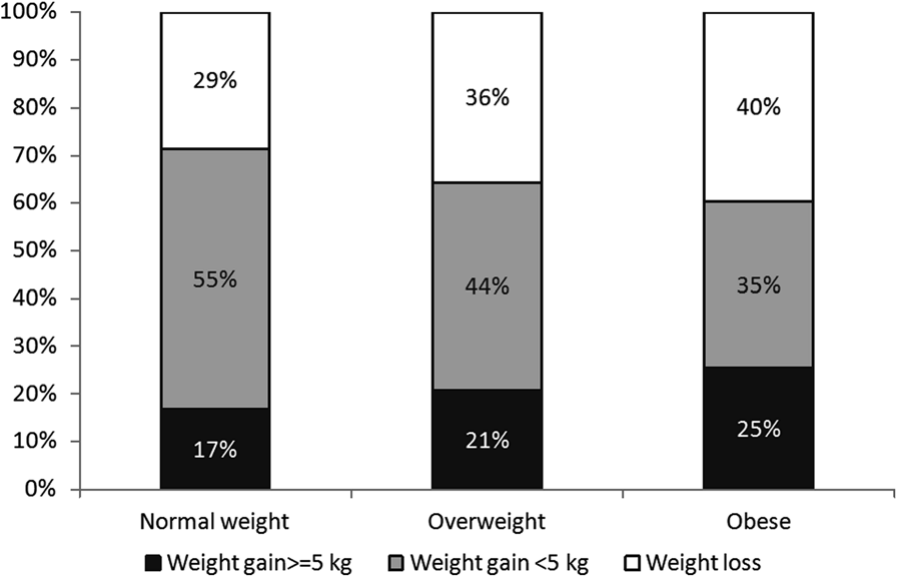
**Proportion of normal weight, overweight and obese participants who had a weight gain ≥5 kg, 0 ≤ . <5 kg and a weight loss between baseline and follow-up (N = 4469).**

The fact that younger people tend to gain more weight is in accordance with other studies [13]. This can probably be explained by the ceiling phenomenon of weight gain through life, which means that because older people are heavier, they are less likely to gain weight. No difference in weight gain was found between normal weight, overweight and obese participants; conversely, obese participants had an increased likelihood of gaining ≥5 kg. A possible explanation is that besides the substantial group of obese patients with weight gain ≥5 kg, there was also a large group of obese patients with weight loss, most likely due to medical reasons. Indeed, the prevalence of obese subjects with weight loss (40%) was higher than in overweight (36%) or normal weight (29%) participants (<0.001). Our results thus suggest that many obese participants tend to lose weight, but that this trend is overcompensated by a significant fraction of obese participants who gained more than 5 kg during the study period. Also, many normal weight or overweight participants tend to gain weight, but less than 5 kg (Figure 1).

Being married (and particularly the marital transition to being married) has been associated with an increase in body weight in most [3,14,15] but not all [16] studies. In this study, living in couple was strongly protective against weight gain, and adjusting for other family covariates such as having children did not change the results (adjusted mean ± sem: 0.36 ± 0.02 vs. 0.29 ± 0.02 kg/year for living alone vs. living in couple, p < 0.05). A possible explanation is the positive effect of living in couple on health-related behaviours [17,18], but further studies are needed to better assess this point.

Smoking at baseline was positively associated with weight gain, and this association persisted after multivariable adjustment. A possible explanation is that some smokers quit during follow-up, which led to increased weight [19], although this statement has been challenged [20]. Other explanations include a less healthy lifestyle of smokers (i.e. less healthy eating and less physical activity) and also the positive association between number of cigarettes smoked and central fat accumulation [21] and a J- or U-shaped association has also been found between waist circumference and visceral fat area with total lifetime smoking amount [22]. Hence, smoking should not be considered as a method for weight maintenance, and the beneficial effects of smoking cessation on health largely overcome the effects of post-cessation weight gain [23].

Receiving social help was associated with an increased likelihood of gaining ≥5 kg over the follow up. In Switzerland, social help is provided as financial support to people with disabilities or whose income is insufficient to support themselves or their family, and can thus be considered as an indicator of financial adversity. The association between receiving social help and weight gain was only partially attenuated after multivariable adjustment. Similar to other studies [5], our results suggest that even in a wealthy country like Switzerland, financial difficulties are positively associated with weight gain, and that this association is independent from educational level. One possible explanation is that financial difficulties might prompt a decrease in diet quality [24], but this aspect requires to be further investigated.

Low educational level has been shown to be associated with increased weight gain [5,25,26]. In this study, no significant association between education and weight gain was found, although participants with university education tended to be at lower risk of weight gain. A possible explanation might be a low statistical power due to a small sample size. Also, given the large educational differences in obesity at baseline [9], ceiling effects may be at play, with people in the lowest educational category already having reached a plateau in obesity prevalence.

### Strengths and limitations

The main strengths of this study are the use of prospective data and of objective anthropometric measurements. This study also has limitations. First, 25% of participants at baseline were not followed-up and were thus not included in this analysis. Participants who accepted to be followed were significantly younger, more educated, more frequently born in Switzerland, received social help less frequently, were less frequently smokers and more frequently active than participants who refused follow-up (Additional file 2: Table S2). Second, no information was available regarding slimming diets at baseline, so it was not possible to adjust for this covariate. Third, a considerable number of participants were foreigners, and education categorization might differ according to the educational system. However, to reduce this issue, we used broad categorizations of education, and almost all foreigners came from European countries, where the educational system is quite comparable. Finally, changes in some sociodemographic or behavioural factors during follow-up might have influenced weight gain. Still, taking into account the multiple possibilities (for instance, physical activity would be split in four groups depending on baseline and follow-up status) would considerably complicate the model and increase the risk of small sized groups, leading to nonsignificant associations due to large confidence intervals of the estimators. Thus, and also considering the relative short follow-up period, we chose to apply a classic analytical method, taking into account only the baseline data.

### Policy implications

Our results are important for public health professionals and policy makers for several reasons. First, being physically active was negatively associated with weight gain, supporting the importance of promoting physical activity, also through environments favouring the practice of physical activity [27,28]. Second, participants with financial difficulties had a higher tendency to gain weight over the follow-up, probably due to their intake of caloric-dense, less expensive foods [29-31]. Importantly, several randomized controlled trials have shown that education alone does not impact the purchase of healthy foods, and that cost reduction and/or promotions are needed to increase fruit and vegetable intake [32-34]. Thus, efforts should be made in the promotion of healthy eating, namely by decreasing the costs of healthy foods rather than just implementing food education campaigns. Finally, the fact that current smokers have an increased risk of gaining over 5 kg could be used as an additional argument for prompting smoking cessation.

## Conclusions

In Switzerland, financial difficulties and current smoking are positively associated with weight gain and living in couple, being older or physically active are negatively associated with weight gain.

## Additional files

Additional file 1: Table S1. Multivariate analysis of the factors associated with weight gain (N = 4,997), including participants reporting involuntary weight loss. Additional file 2: Table S2. Comparison of the baseline characteristics between participants who refused (N=1573) and who accepted (N = 4968) follow-up.
