# Supplementary material for: Socio-demographic and behavioural determinants of weight gain in the Swiss population
Source: BMC Public Health. 2015 Jan 31;15:73. doi: 10.1186/s12889-015-1451-9 (PMC4320497; doi:10.1186/s12889-015-1451-9)
Supplement: Additional file 1: Table S1. — Multivariate analysis of the factors associated with weight gain (N = 4,997), including participants reporting involuntary weight loss. [file 12889_2015_1451_MOESM1_ESM.doc]

Supplementary table 1: Multivariate analysis of the factors associated with weight gain (N=4,997), including participants reporting involuntary weight loss.

|  | Weight gain (kg/year) | | | Gain≥5 kg | | |
| --- | --- | --- | --- | --- | --- | --- |
|  | Model 1 | Model 2 | Model 2 | Model 1 | Model 2 | Model 2 |
| Age group |  |  |  |  |  |  |
| [35-45[ | 0.43 ± 0.02 | 0.42 ± 0.02 | 0.43 ± 0.02 | 1 (ref.) | 1 (ref.) | 1 (ref.) |
| [45-55[ | 0.28 ± 0.02 | 0.27 ± 0.02 | 0.27 ± 0.02 | 0.78 (0.65 - 0.93) | 0.74 (0.62 - 0.88) | 0.72 (0.60 - 0.87) |
| [55-65[ | 0.14 ± 0.02 | 0.15 ± 0.02 | 0.15 ± 0.02 | 0.62 (0.51 - 0.75) | 0.56 (0.46 - 0.68) | 0.54 (0.44 - 0.66) |
| [65-75] | -0.06 ± 0.03 | -0.04 ± 0.04 | -0.05 ± 0.04 | 0.34 (0.26 - 0.46) | 0.33 (0.24 - 0.44) | 0.31 (0.23 - 0.42) |
| p-value between groups | <0.001 ^a^ | <0.001 | <0.001 | <0.001 ^d^ | <0.001 ^d^ | <0.001 ^d^ |
| Gender |  |  |  |  |  |  |
| Women | 0.25 ± 0.02 | 0.24 ± 0.02 | 0.25 ± 0.02 | 1 (ref.) | 1 (ref.) | 1 (ref.) |
| Men | 0.24 ± 0.02 | 0.25 ± 0.02 | 0.24 ± 0.02 | 1.00 (0.86 - 1.16) | 0.95 (0.82 - 1.11) | 1.05 (0.91 - 1.23) |
| p-value between groups | 0.65 ^b^ | 0.70 | 0.93 | 0.88 | 0.48 | 0.49 |
| Born in Switzerland |  |  |  |  |  |  |
| No | 0.26 ± 0.02 | 0.27 ± 0.02 | - | 1 (ref.) | 1 (ref.) | - |
| Yes | 0.24 ± 0.02 | 0.23 ± 0.02 | - | 0.98 (0.84 - 1.13) | 1.04 (0.89 - 1.21) | - |
| p-value between groups | 0.30 | 0.23 | - | 0.76 | 0.62 |  |
| Nationality |  |  |  |  |  |  |
| Swiss | 0.24 ± 0.02 | - | 0.24 ± 0.02 | 1 (ref.) | - | 1 (ref.) |
| French | 0.27 ± 0.05 | - | 0.26 ± 0.05 | 0.97 (0.71 - 1.31) | - | 0.98 (0.72 - 1.34) |
| Italian | 0.36 ± 0.05 | - | 0.36 ± 0.05 | 1.35 (0.99 - 1.83) | - | 1.30 (0.95 - 1.78) |
| Portuguese | 0.11 ± 0.06 | - | 0.12 ± 0.06 | 0.68 (0.47 - 0.97) | - | 0.66 (0.46 - 0.95) |
| Spanish | 0.27 ± 0.07 | - | 0.27 ± 0.07 | 1.06 (0.72 - 1.56) | - | 1.06 (0.72 - 1.57) |
| Other | 0.27 ± 0.03 | - | 0.27 ± 0.03 | 1.06 (0.87 - 1.29) | - | 1.02 (0.83 - 1.24) |
| p-value between groups | 0.05 | - | 0.05 | - |  | - |
| Receiving social help |  |  |  |  |  |  |
| No | 0.25 ± 0.01 | 0.25 ± 0.01 | 0.25 ± 0.01 | 1 (ref.) | 1 (ref.) | 1 (ref.) |
| Yes | 0.23 ± 0.04 | 0.22 ± 0.04 | 0.23 ± 0.04 | 1.35 (1.10 - 1.65) | 1.18 (0.96 - 1.46) | 1.22 (0.99 - 1.51) |
| p-value between groups | 0.72 | 0.54 | 0.59 | <0.005 | 0.11 | 0.06 |
| Marital status |  |  |  |  |  |  |
| Living alone | 0.30 ± 0.02 | 0.30 ± 0.02 | 0.29 ± 0.02 | 1 (ref.) | 1 (ref.) | 1 (ref.) |
| Living in couple | 0.22 ± 0.02 | 0.22 ± 0.02 | 0.22 ± 0.02 | 0.71 (0.61 - 0.83) | 0.74 (0.63 - 0.86) | 0.75 (0.64 - 0.87) |
| p-value between groups | 0.006 | 0.009 | 0.01 | <0.001 | <0.001 | <0.001 |
| Educational level |  |  |  |  |  |  |
| Primary | 0.24 ± 0.03 | - | - | 1 (ref.) | - | - |
| Apprenticeship | 0.24 ± 0.02 | - | - | 1.04 (0.84 - 1.29) | - | - |
| Secondary school | 0.25 ± 0.02 | - | - | 0.91 (0.73 - 1.14) | - | - |
| University | 0.25 ± 0.03 | - | - | 0.85 (0.67 - 1.08) | - | - |
| p-value between groups | 0.99 | - | - | 0.07 |  |  |
| Smoking status |  |  |  |  |  |  |
| Never | 0.24 ± 0.02 | 0.24 ± 0.02 | 0.24 ± 0.02 | 1 (ref.) | 1 (ref.) | 1 (ref.) |
| Former | 0.22 ± 0.02 | 0.22 ± 0.02 | 0.22 ± 0.02 | 1.10 (0.92 - 1.31) | 1.09 (0.91 - 1.30) | 1.08 (0.90 - 1.29) |
| Current | 0.30 ± 0.02 | 0.29 ± 0.03 | 0.29 ± 0.03 | 1.52 (1.27 - 1.81) | 1.48 (1.24 - 1.78) | 1.45 (1.21 - 1.73) |
| p-value between groups | 0.043 | 0.13 | 0.11 | <0.001 ^d^ | <0.001 ^d^ | <0.001 ^d^ |
| Physical activity |  |  |  |  |  |  |
| No | 0.26 ± 0.02 | 0.26 ± 0.02 | 0.26 ± 0.02 | 1 (ref.) | 1 (ref.) | 1 (ref.) |
| Yes | 0.23 ± 0.02 | 0.23 ± 0.02 | 0.23 ± 0.02 | 0.75 (0.65 - 0.86) | 0.83 (0.71 - 0.96) | 0.80 (0.69 - 0.94) |
| p-value between groups | 0.35 | 0.32 | 0.34 | <0.001 | 0.02 | 0.005 |
| Body mass index categories |  |  |  |  |  |  |
| Normal | 0.27 ± 0.02 | 0.27 ± 0.02 | - | 1 (ref.) | 1 (ref.) | - |
| Overweight | 0.24 ± 0.02 | 0.24 ± 0.02 | - | 1.48 (1.26 - 1.75) | 1.47 (1.24 - 1.74) | - |
| Obese | 0.17 ± 0.03 | 0.17 ± 0.03 | - | 2.01 (1.63 - 2.49) | 1.98 (1.59 - 2.47) | - |
| p-value between groups | <0.05 | <.05 | - | <0.001 ^d^ | <0.001 ^d^ |  |
| Abdominal obesity |  |  |  |  |  |  |
| No | 0.26 ± 0.01 | - | 0.27 ± 0.01 | 1 (ref.) | - | 1 (ref.) |
| Yes | 0.20 ± 0.02 | - | 0.19 ± 0.02 | 1.59 (1.35 - 1.87) | - | 1.54 (1.30 - 1.82) |
| p-value between groups | <0.05 | - | <0.05 | <0.001 |  | <0.001 |

OR: Odds Ratio; BMI: Body Mass Index. Results are expressed as multivariate adjusted mean ± standard error of the mean (sem) or as odds-ratio (OR) and (95% CI). Statistical analysis by analysis of variance or logistic regression. Model 1, adjusting for age and gender, except ^a^ adjusted for gender only; ^b^ adjusted for age only; Model 2, adjusted for all the variables in the model (indicated in the column); ^d^ p-value of the test for trend; -, not included in the model. Statistically significant (p<0.05) individual ORs are indicated in bold
