# Supplementary material for: Socio-demographic and behavioural determinants of weight gain in the Swiss population
Source: BMC Public Health. 2015 Jan 31;15:73. doi: 10.1186/s12889-015-1451-9 (PMC4320497; doi:10.1186/s12889-015-1451-9)
Supplement: Additional file 2: Table S2. — Comparison of the baseline characteristics between participants who refused (N=1573) and who accepted (N = 4968) follow-up. [file 12889_2015_1451_MOESM2_ESM.doc]

Supplementary table 2: comparison of the baseline characteristics between participants who refused (N=1573) and who accepted (N=4968) follow-up.

|  | Refused | Accepted | p-value |
| --- | --- | --- | --- |
| Age group |  |  |  |
| [35-45[ | 457 (29.1) | 1530 (30.8) |  |
| [45-55[ | 435 (27.7) | 1498 (30.2) | <0.001 |
| [55-65[ | 415 (26.4) | 1303 (26.2) |  |
| [65-75] | 266 (16.9) | 637 (12.8) |  |
| Gender |  |  |  |
| Women | 814 (51.8) | 2663 (53.6) | <0.001 |
| Men | 759 (48.3) | 2305 (46.4) |  |
| Born in Switzerland |  |  |  |
| No | 795 (50.5) | 1851 (37.3) | <0.001 |
| Yes | 778 (49.5) | 3117 (62.7) |  |
| Nationality |  |  |  |
| Swiss | 778 (49.5) | 3117 (62.7) |  |
| French | 120 (7.6) | 314 (6.3) |  |
| Italian | 117 (7.4) | 272 (5.5) | <0.001 |
| Portuguese | 137 (8.7) | 250 (5) |  |
| Spanish | 81 (5.2) | 175 (3.5) |  |
| Other | 340 (21.6) | 840 (16.9) |  |
| Receiving social help |  |  |  |
| No | 1277 (81.2) | 4341 (87.4) | <0.001 |
| Yes | 296 (18.8) | 627 (12.6) |  |
| Marital status |  |  |  |
| Living alone | 544 (34.7) | 1593 (32.1) | 0.06 |
| Living in couple | 1026 (65.4) | 3372 (67.9) |  |
| Educational level |  |  |  |
| Basic | 498 (31.7) | 859 (17.3) |  |
| Apprenticeship | 542 (34.5) | 1760 (35.4) | <0.001 |
| Secondary school | 304 (19.3) | 1284 (25.9) |  |
| University | 229 (14.6) | 1065 (21.4) |  |
| Smoking status |  |  |  |
| Never | 668 (42.5) | 2031 (40.9) |  |
| Former | 445 (28.3) | 1665 (33.5) | <0.001 |
| Current | 460 (29.2) | 1272 (25.6) |  |
| Physical activity |  |  |  |
| No | 850 (54.0) | 2266 (45.6) | <0.001 |
| Yes | 723 (46.0) | 2702 (54.4) |  |
| BMI categories |  |  |  |
| Normal | 677 (43.1) | 2475 (49.8) |  |
| Overweight | 579 (36.9) | 1817 (36.6) | <0.001 |
| Obese | 314 (20.0) | 676 (13.6) |  |
| Abdominal obesity |  |  |  |
| No | 1036 (66.1) | 3599 (72.4) | <0.001 |
| Yes | 531 (33.9) | 1369 (27.6) |  |

Results are expressed as number of participants and (%) after excluding 192 participants who died during follow-up. Statistical comparison using Chi-square.
